# ASC Deficiency Compromises Skeletal Architecture and Mechanical Competence in Mice

**DOI:** 10.3390/bioengineering13101211

**Published:** 2026-10-17

**Authors:** Qilin Pei, Yutao He, Yun Bai, Lingxia He, Xi Li, Bin Wang, Fengning Chuan

**Affiliations:** 1 Department of Medical Experimental Technology, School of Basic Medicine, Chongqing Medical University, Chongqing 400016, China; 2 Department of Endocrinology, Chongqing University Fuling Hospital, Chongqing 408099, China

**Keywords:** ASC, bone microarchitecture, cortical bone geometry, osteocyte lacunar–canalicular system (LCS), bone mechanical properties

## Abstract

Apoptosis-associated speck-like protein containing a caspase recruitment domain (ASC) is a key adaptor of canonical inflammasome signaling and has been implicated in osteoblast differentiation and bone formation. However, whether ASC deficiency alters the structural and mechanical properties of the intact skeleton remains unclear. In this study, the skeletal phenotype of 9-week-old male *Asc*-homozygous-deficient (*Asc^−/−^*) mice was characterized using micro-computed tomography, three-point bending, tibial microindentation, and silver staining of the osteocyte lacunar–canalicular system (LCS). Trabecular microarchitecture, cortical geometry, whole-bone mechanical behavior, tissue-level hardness, and LCS morphology were quantified. Associations between cortical geometric parameters and whole-bone mechanical outcomes were assessed by Pearson correlation analysis. *Asc^−/−^* mice had shorter femora despite greater body weight. Trabecular bone volume fraction, thickness, and number were reduced, whereas trabecular separation was increased. Cortical analysis showed reductions in total cross-sectional area, cortical area, cortical thickness, periosteal perimeter, minimum principal moment of inertia, and polar moment of inertia. Femoral maximum load, stiffness, yield force, and work to fracture were reduced, together with lower tibial cortical hardness. ASC deficiency was also associated with enlarged osteocyte lacunae, reduced canalicular length and density, and increased canalicular width. Cortical area, thickness, and moment of inertia showed strong positive associations with whole-bone mechanical outcomes. Overall, ASC deficiency is associated with a distinct skeletal phenotype characterized by impaired trabecular architecture, reduced cortical size and load-bearing geometry, decreased whole-bone mechanical competence, lower tissue-level hardness, and altered osteocyte LCS organization. These findings extend the known skeletal role of ASC beyond osteogenesis and bone repair to the structural and mechanical phenotype of the intact skeleton.

## 1. Introduction

Bone is a dynamic and adaptable organ that actively helps keep our body balanced in response to mechanical forces [1]. Bone mechanical function depends not only on bone mass but also on tissue microarchitecture, spatial organization, and material properties [2,3]. Trabecular microarchitecture affects the mechanical behavior of cancellous bone by controlling trabecular connectivity and load sharing. Meanwhile, cortical area, thickness, and cross-sectional shape directly influence the ability of long bones to withstand bending forces and external loads [4,5]. Changes in bone microarchitecture and geometry can weaken overall bone strength [6]. Therefore, an integrated assessment of bone microarchitecture, cross-sectional geometry, and mechanical behavior is important for defining the skeletal consequences of genetic perturbation.

Bone homeostasis is maintained by a balance between bone formation and resorption, which is regulated by multiple cellular signaling pathways and inflammatory mechanisms [7,8,9]. Disruption of this balance can result in skeletal deterioration and reduced bone strength. Growing evidence implicates inflammasome signaling in bone formation, remodeling, and repair. Apoptosis-associated speck-like protein containing a caspase recruitment domain (ASC), encoded by the *Pycard* gene, is a key adaptor protein required for the assembly of several canonical inflammasomes [10]. Beyond its established role in inflammatory signaling, ASC has been implicated in skeletal cell function. Previous studies showed that ASC deficiency impaired BMP7-induced osteoblast differentiation and delayed bone formation during defect healing [11]. ASC deficiency has also been associated with reduced new bone deposition in response to bone substitute materials [12]. These studies established a role for ASC in osteoblast differentiation and bone formation under regenerative or experimental conditions. However, whether loss of ASC alters the architecture and mechanical competence of the intact skeleton remains poorly defined.

Whole-bone mechanical behavior reflects the combined influence of bone size, cross-sectional geometry, and local tissue properties [13,14,15,16]. Growing evidence shows that the osteocyte lacunar–canalicular system (LCS) within the bone matrix acts as the main mechanosensory network, crucial for determining the material quality and mechanical behavior of bone tissue [17,18]. Disruption of the LCS is associated with impaired bone microarchitecture, reduced bone quality, and increased skeletal fragility [19]. Thus, examining the LCS together with cortical geometry and mechanical behavior may provide additional insight into the skeletal phenotype associated with ASC deficiency. Given the established effects of ASC deficiency on osteoblast-lineage cells and bone formation, we therefore hypothesized that ASC deficiency would also be associated with alterations in osteocyte LCS organization.

In this study, we characterized the structural and mechanical phenotypes of the skeleton in male *Asc*-homozygous-deficient (*Asc^−/−^*) mice. Femoral trabecular microarchitecture and cortical geometry were quantified by micro-computed tomography, and whole-bone mechanical behavior was evaluated by three-point bending. Tibial microindentation was used as a complementary assessment of tissue-level hardness. Osteocyte LCS morphology was examined in cortical bone. We further analyzed the relationships between cortical geometry and whole-bone mechanical outcomes. This approach was used to determine whether ASC deficiency is associated with coordinated alterations in skeletal architecture, load-bearing geometry, and mechanical competence in the absence of induced bone injury.

## 2. Materials and Methods

### 2.1. Animals

*Asc^−^*^/^*^−^* mice were generously provided by Dr. Lu and Dr. Zong from the Second Military Medical University. Nine-week-old male *Asc^−^*^/^*^−^* mice and age-matched C57BL/6J (WT) mice were used in this study. All animals were housed at 22 ± 2 °C in standard cages with a 12-h light/dark cycle and ad libitum access to sterile food and water. Body weights were recorded prior to euthanasia. Subsequently, the femora, tibiae, and humeri were harvested and meticulously cleared of all surrounding soft tissues. All animal procedures were approved by the Institutional Animal Care and Use Committee of Chongqing Medical University.

### 2.2. Western Blotting

Mouse tails were collected and homogenized in lysis buffer supplemented with protease and phosphatase inhibitor cocktails. The extracted proteins were separated by SDS–polyacrylamide gel electrophoresis (SDS-PAGE) and subsequently transferred onto polyvinylidene difluoride (PVDF) membranes. After blocking with 5% skim milk for 1 h at room temperature, the membranes were incubated overnight at 4 °C with primary antibodies against ASC (1:1000, #67824S, Cell Signaling Technology, Danvers, MA, USA) and β-actin (1:1000, #4967S, Cell Signaling Technology, Danvers, MA, USA). Subsequently, the membranes were incubated with horseradish peroxidase (HRP)-conjugated anti-rabbit secondary antibodies for 1 h at room temperature. Protein signals were detected using an enhanced chemiluminescence (ECL) detection system. Western blotting was performed using tail samples from four mice per group. β-actin was used as a loading control to verify comparable protein loading among samples. Because the analysis was performed to confirm loss of detectable ASC protein expression in *Asc^−^*^/^*^−^* mice rather than to quantify a graded change in ASC expression, densitometric normalization of ASC to β-actin was not performed.

### 2.3. Micro-CT Analysis

The left femora (*n* = 5/group) were scanned with a SkyScan 1076 micro-computed tomography system (Bruker microCT, Kontich, Belgium) at 29 kV and 175 μA, with an exposure time of 900 ms and a rotation step of 0.3°. No filter was used. Images were acquired at a voxel size of 9.13 μm and reconstructed with NRecon software (version 1.7.1.0, Bruker microCT) using a smoothing setting of 3, a ring-artifact correction of 3, and a beam-hardening correction of 30%. Reconstructed images were reoriented with DataViewer (version 1.5.6, Bruker microCT) and analyzed with CTAn software (Bruker microCT). Micro-CT analysis was performed with investigators blinded to genotype.

Trabecular bone microarchitecture was assessed in the distal femoral metaphysis over 201 consecutive transverse slices, corresponding to an approximately 1.84-mm region. Cortical geometry was analyzed at the femoral mid-diaphysis over 51 consecutive transverse slices, corresponding to an approximately 0.47-mm region. Trabecular parameters included bone volume fraction (BV/TV, %), trabecular thickness (Tb.Th, mm), trabecular number (Tb.N, 1/mm), and trabecular separation (Tb.Sp, mm). Cortical parameters included total cross-sectional area (Tt.Ar, mm^2^), cortical area (Ct.Ar, mm^2^), cortical area fraction (Ct.Ar/Tt.Ar, %), cortical thickness (Ct.Th, mm), periosteal perimeter (Ps.Pm, mm), endocortical perimeter (Ec.Pm, mm), polar moment of inertia (pMOI, mm^4^), and minimum principal moment of inertia (*I*_min_, mm^4^).

### 2.4. Serum Bone Turnover Markers

Serum samples from five mice per group (*n* = 5/group) were isolated from whole blood by centrifugation. Serum procollagen type I N-terminal propeptide (P1NP) and tartrate-resistant acid phosphatase 5b (TRAcP5b) concentrations were measured using commercially available mouse ELISA kits (Shanghai BlueGene Biotech Co., Ltd., Shanghai, China) according to the manufacturer’s instructions.

### 2.5. Mechanical Testing

Following micro-CT, femora (*n* = 5/group) were subjected to three-point bending. Femora were stored at −20 °C in saline-soaked gauze, fully thawed before testing, and tested in a hydrated state. Each femur was positioned on two supports 7 mm apart, with the anterior surface facing the loading nose. After a preload of 0.1 N, specimens were loaded at 0.05 mm/s until fracture. We recorded load–displacement curves and obtained mechanical parameters, including maximum load (F, N), stiffness (K, N/mm), yield force (N), work to fracture (Nmm), and post-yield displacement (mm). Following established guidelines [14], elastic modulus (*E*, GPa) was estimated using beam theory as:

(1) $$E=\frac{KL^{3}}{48I_{\min}}$$

where K is bending stiffness, L is the support span, and *I*_min_ is the minimum principal moment of inertia of the corresponding femur.

Tibial cortical tissue-level mechanical properties were assessed by Vickers microindentation in five mice per group (*n* = 5/group). Transverse sections approximately 1 mm thick were prepared from the tibial mid-diaphysis and polished to obtain a smooth testing surface. Ten non-overlapping indentations were made within the cortical bone matrix of each specimen using a 50-g load. Vickers hardness (HV) was calculated from the indentation dimensions as previously described [20]. The mean value of the 10 indentations was used as the representative HV value for each animal. The microindentation-derived Young’s modulus (*E_i_*) and yield stress (*σ_y_*) were subsequently calculated using previously reported empirical relationships [21]:

(2) *E_i_* = 0.36*HV* + 0.58,

(3) *σ_y_* = 2.12*HV* + 8.16,

Three-point bending and microindentation were performed with investigators blinded to genotype.

### 2.6. Histological Staining and Bone Histomorphometric Analysis

Right femora (*n* = 5/group) were fixed in 4% paraformaldehyde for 24 h and decalcified in 14% EDTA at 4 °C for 3 weeks. After decalcification, specimens were embedded in paraffin and cut into 5-μm-thick sections. Sections were stained with hematoxylin and eosin (H&E) for evaluation and quantification of osteoblast-related parameters (including Ob.S/B.Pm and N.Ob/B.Pm), while tartrate-resistant acid phosphatase (TRAP) staining was performed to identify and quantify osteoclasts (including Oc.S/B.Pm and N.Oc/B.Pm).

To visualize and quantify the osteocyte LCS, sections were stained with silver nitrate. Images were acquired at 100 × magnification from five non-overlapping fields in the femoral mid-diaphyseal cortex for each mouse. Grayscale images were converted to binary images using an automatic threshold function in ImageJ (version 1.53v) for segmentation of silver-stained lacunae and canaliculi. Lacunae with a length-to-width ratio < 2.5 were included in the analysis. Approximately 30–40 lacunae and 200–400 canaliculi were analyzed per mouse. All clearly identifiable canaliculi extending from the selected lacunae were included for canalicular measurements. LCS parameters included lacunar area, canalicular length, canalicular density, and mean canalicular width. For canalicular width measurements, images were further processed using the Sharpen and Find Edges functions in ImageJ, and grayscale intensity profiles were used to define the canalicular boundaries according to a previously described method [22]. Measurements from the five fields were averaged to obtain one value for each mouse (*n* = 5 per group). Histomorphometric and LCS analyses were performed in a blinded manner.

### 2.7. Statistical Analysis

Statistical analyses were performed using GraphPad Prism 10.1 (GraphPad Software). Data are presented as mean ± SD. Differences between two groups were assessed using a two-tailed unpaired *t*-test with Welch’s correction. Pearson correlation coefficients were calculated between eight micro-CT-derived cortical geometric parameters (Ct.Ar, Tt.Ar, Ct.Ar/Tt.Ar, Ct.Th, Ps.Pm, Ec.Pm, pMOI, and *I*_min_) and five whole-bone mechanical outcomes (maximum load, stiffness, yield force, work to fracture, and post-yield displacement) using pooled data from WT and *Asc^−/−^* mice (*n* = 10). *p* values from the 40 correlation tests were adjusted for multiple testing using the Benjamini–Hochberg false discovery rate (FDR) procedure. Estimated elastic modulus was not included in the correlation analysis because it was derived from stiffness and *I*_min_. Statistical significance was defined as *p* < 0.05 for group comparisons and FDR-adjusted *p* < 0.05 for correlation analyses.

## 3. Results

### 3.1. Validation of Asc^−/−^ Mice and Skeletal Phenotype Characterization

**Figure 1:**
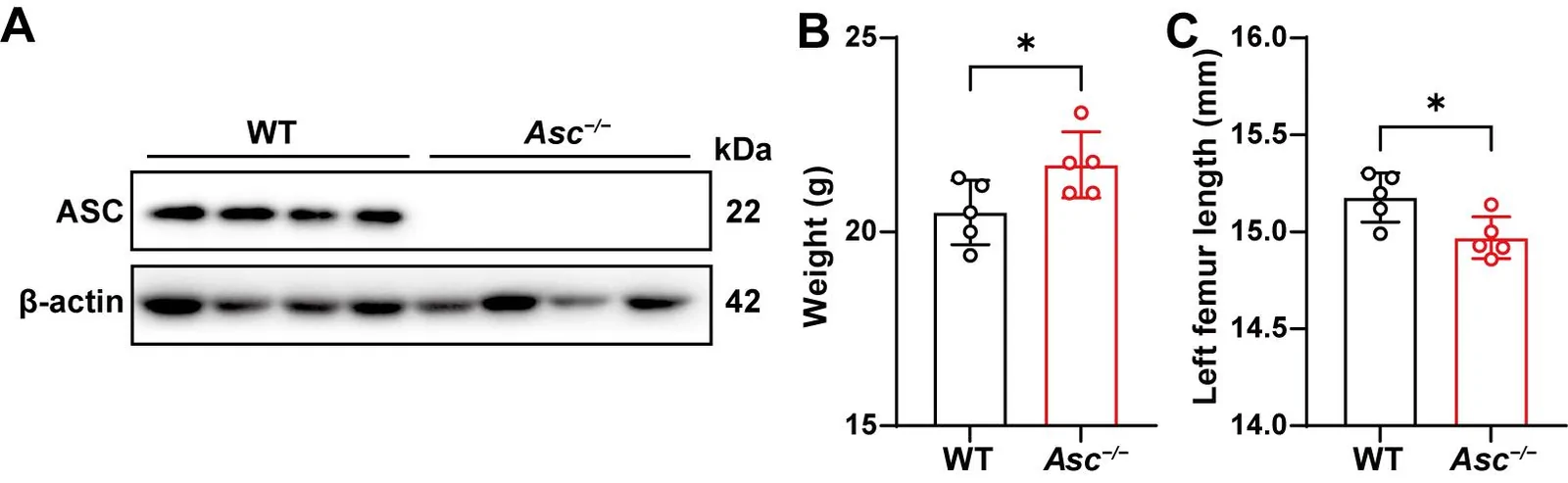
Validation of *Asc*^−/−^ mice and skeletal phenotype characterization. (**A**) Western blot analysis of ASC protein in mouse tail (*n* = 4 /group). (**B**) Body weight of WT and *Asc*^−/−^ mice (*n* = 5/group). (**C**) Quantitative measurement of left femur length in WT and *Asc*^−/−^ mice (*n* = 5/group). Individual points represent biological replicates; data are presented as mean ± SD. Differences between groups were analyzed using a two-tailed unpaired *t*-test with Welch’s correction. * *p* < 0.05.

To investigate ASC function in bone, we first characterized *Asc*^−/−^ mice and compared them with age-matched WT mice. Western blotting detected ASC protein in WT tail tissues, whereas no detectable ASC signal was observed in *Asc*^−/−^ mice (Figure 1A), confirming loss of ASC protein expression. We next examined the physical characteristics of the animals. Interestingly, *Asc*^−/−^ mice exhibited a significant increase in body weight compared with WT mice (Figure 1B). However, quantitative measurement revealed a marked reduction in left femoral length in *Asc*^−/−^ mice (Figure 1C). This combination of increased body mass and reduced femoral length indicates a difference in overall skeletal size in *Asc*^−/−^ mice.

### 3.2. ASC Deficiency Impairs Trabecular and Cortical Bone Architecture

**Figure 2:**
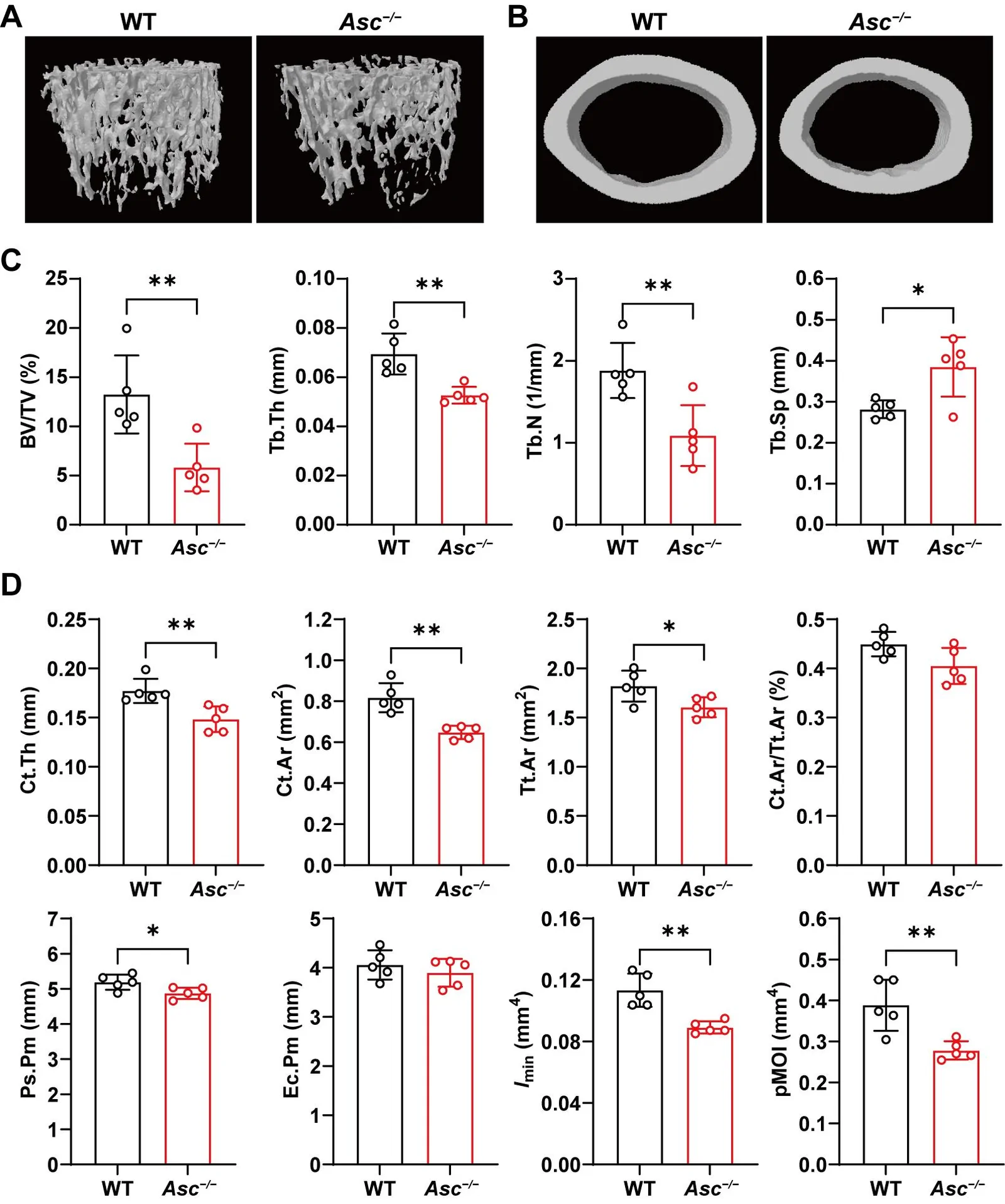
ASC deficiency impairs trabecular and cortical bone architecture. (**A**) Representative 3D micro-CT reconstruction images of femoral trabecular bone microarchitecture. (**B**) Representative 3D micro-CT reconstruction images of mid-diaphyseal cortical bone. (**C**) Quantification of trabecular bone parameters: bone volume fraction (BV/TV), trabecular thickness (Tb. Th), trabecular number (Tb. N) and trabecular separation (Tb. Sp). (**D**) Quantification of cortical bone parameters: cortical thickness (Ct.Th), cortical area (Ct.Ar), total cross-sectional area (Tt.Ar), cortical area fraction (Ct.Ar/Tt.Ar), periosteal perimeter (Ps.Pm), endocortical perimeter (Ec.Pm), minimum principal moment of inertia (*I*_min_), and polar moment of inertia (pMOI). *n* = 5 mice per group. Individual points represent biological replicates, and data are presented as mean ± SD. Differences between groups were analyzed using a two-tailed unpaired *t*-test with Welch’s correction. * *p* < 0.05, ** *p* < 0.01.

Micro-CT analysis revealed marked changes in both trabecular and cortical bone in *Asc*^−/−^ mice (Figure 2). Quantitative analysis of trabecular bone parameters showed that *Asc*^−/−^ mice exhibited significant reductions in trabecular bone volume fraction (BV/TV), trabecular number (Tb.N), and trabecular thickness (Tb.Th), accompanied by increased trabecular separation (Tb.Sp) (Figure 2A,C). Histological analysis of the trabecular bone surface further revealed reduced osteoblast surface and number (Ob.S/B.Pm and N.Ob/B.Pm), together with increased osteoclast surface and number (Oc.S/B.Pm and N.Oc/B.Pm) in *Asc*^−/−^ mice compared with WT mice (Figure S1A–D). Consistent with these histological findings, serum P1NP was decreased and TRAcP5b was increased in *Asc*^−/−^ mice.

Analysis of cortical bone further revealed significant decreases in total cross-sectional area (Tt.Ar), cortical area (Ct.Ar), cortical thickness (Ct.Th), and periosteal perimeter (Ps.Pm) in *Asc*^−/−^ mice. In addition, cortical geometric parameters related to mechanical properties, including the polar moment of inertia (pMOI) and minimum principal moment of inertia (*I*_min_), were significantly reduced in *Asc*^−/−^ mice (Figure 2B,D). These findings show that ASC deficiency deteriorates both trabecular microarchitecture and cortical bone geometry, indicating impaired skeletal structural integrity.

### 3.3. Asc^−/−^ Mice Exhibit Reduced Whole-Bone and Tissue-Level Mechanical Properties

**Figure 3:**
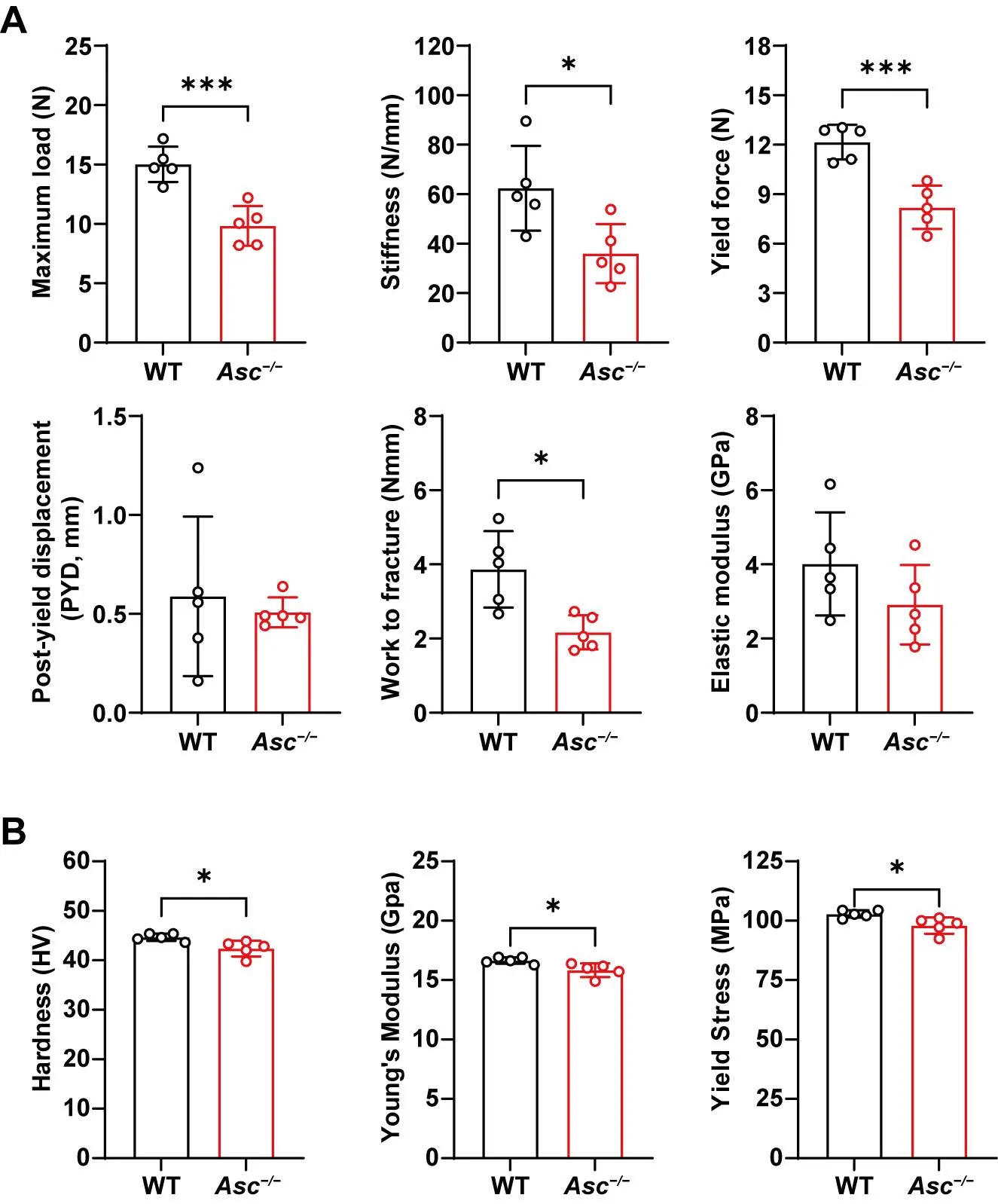
ASC deficiency is associated with impaired whole-bone and tissue-level mechanical properties. (**A**) Whole-bone mechanical properties measured by three-point bending, including maximum load, stiffness, yield force, work to fracture, post-yield displacement (PYD), and estimated elastic modulus. (**B**) Tibial cortical matrix properties assessed by microindentation, including hardness, Young’s modulus, and yield stress. *n* = 5 mice per group. Individual points represent biological replicates, and data are presented as mean ± SD. Differences between groups were analyzed using a two-tailed unpaired *t*-test with Welch’s correction. * *p* < 0.05, *** *p* < 0.001.

Whole-bone mechanical properties were measured using a three-point bending test (Figure 3A). Compared with WT mice, *Asc*^−/−^ mice exhibited significantly reduced maximum load, stiffness, yield force, and work to fracture, whereas post-yield displacement and estimated elastic modulus did not differ significantly between groups. Microindentation analysis further revealed decreased hardness, Young’s modulus, and yield stress in *Asc*^−/−^ mice (Figure 3B). These findings demonstrate that ASC deficiency impaired bone mechanical properties at both the whole-bone mechanical performance and tissue material properties.

### 3.4. ASC Deficiency Is Associated with Altered Osteocyte Lacunar–Canalicular System Architecture

**Figure 4:**
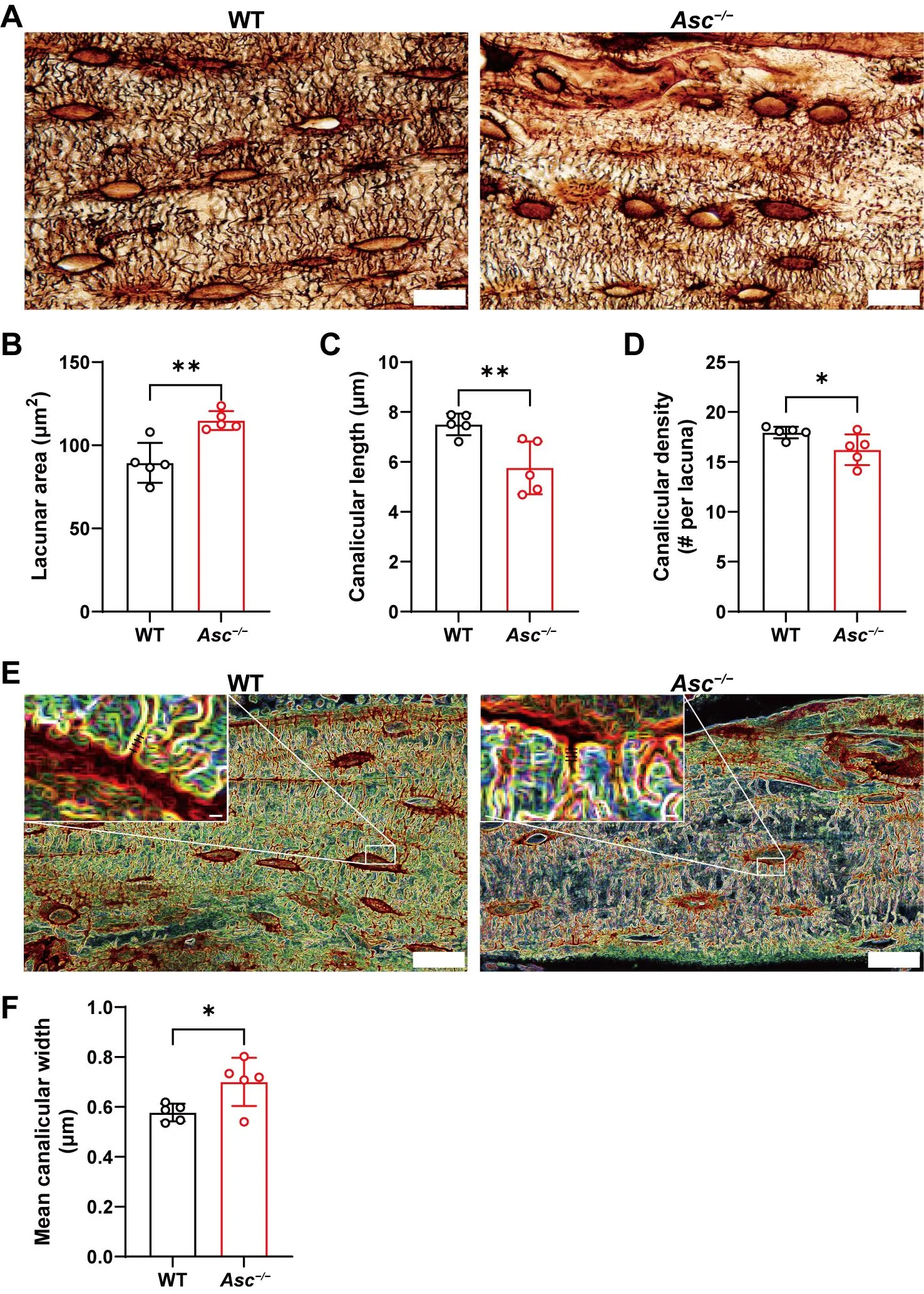
ASC deficiency is associated with altered osteocyte lacunar–canalicular system architecture. (**A**) Representative silver nitrate staining images of the cortical osteocyte lacunar–canalicular system. Scale bar: 20 μm. (**B**–**D**) Quantification of lacunar area (**B**), canalicular length (**C**), and canalicular density (**D**). (**E**) Representative processed images used for canalicular width measurement. Scale bar: 20 μm; inset scale bar: 1 μm. (**F**) Quantification of mean canalicular width. *n* = 5 mice per group. Individual points represent biological replicates, and data are presented as mean ± SD. Differences between groups were analyzed using a two-tailed unpaired *t*-test with Welch’s correction. * *p* < 0.05, ** *p* < 0.01.

Silver nitrate staining was performed to visualize the lacunar–canalicular network in cortical bone. Compared with WT mice, *Asc*^−/−^ mice exhibited apparent abnormalities in LCS architecture, characterized by enlarged osteocyte lacunar area, reduced canalicular length, and decreased canalicular density (Figure 4A–D). In addition, *Asc*^−/−^ mice displayed increased mean canalicular width compared with WT mice (Figure 4E,F). Taken together, these findings show that ASC deficiency is associated with marked alterations in osteocyte LCS architecture.

### 3.5. Relationships Between Cortical Bone Geometry and Whole-Bone Mechanical Properties

**Figure 5:**
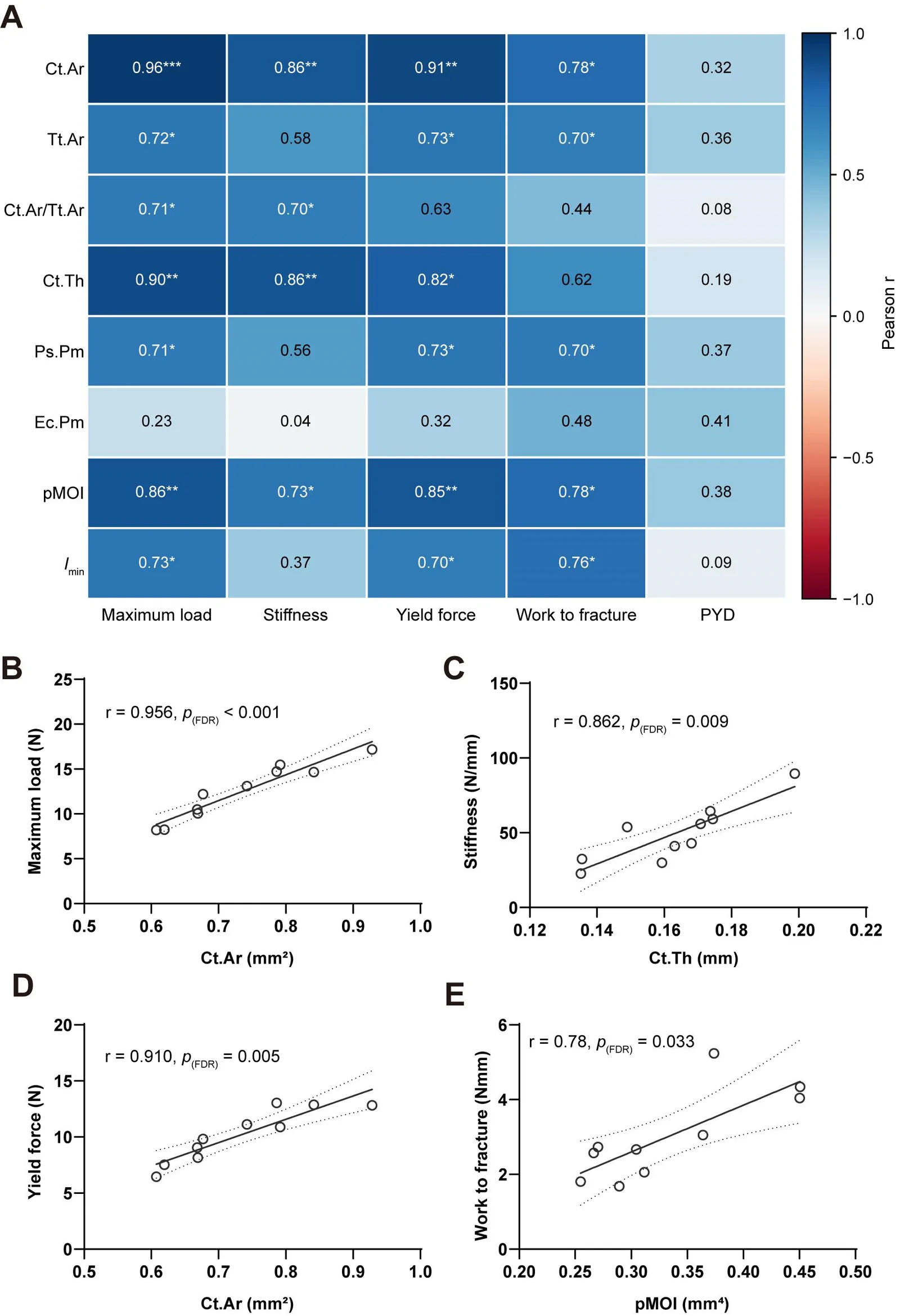
Associations between cortical bone geometry and whole-bone mechanical properties. (**A**) Heatmap showing Pearson correlation coefficients (*r*) between cortical geometric parameters and mechanical outcomes measured by three-point bending, including maximum load, stiffness, yield force, work to fracture, and post-yield displacement (PYD). *p* values from all 40 correlation analyses were adjusted for multiple testing using the Benjamini–Hochberg false discovery rate procedure. Asterisks indicate significance based on FDR-adjusted *p* values: * *p* < 0.05, ** *p* < 0.01, and *** *p* < 0.001. (**B**–**E**) Scatter plots showing the strongest FDR-significant association for each of four representative mechanical outcomes: (**B**) Ct.Ar versus maximum load, (**C**) Ct.Th versus stiffness, (**D**) Ct.Ar versus yield force, and (**E**) pMOI versus work to fracture. Each point represents one mouse (*n* = 10, pooled WT and *Asc*^−/−^ mice). Solid lines indicate the fitted linear regression, and dotted lines indicate the 95% confidence intervals. Pearson *r* and FDR-adjusted *p* values are shown in each panel.

Pearson correlation analysis was performed to evaluate the relationships between cortical geometric parameters and whole-bone mechanical outcomes using pooled data from WT and *Asc*^−/−^ mice (*n* = 10). After Benjamini–Hochberg FDR correction, 22 of the 40 correlations remained statistically significant (Figure 5A). Among the four representative mechanical outcomes, the strongest FDR-significant associations were observed between Ct.Ar and maximum load (*r* = 0.956, FDR-adjusted *p* < 0.001), Ct.Th and stiffness (*r* = 0.862, FDR-adjusted *p* = 0.009), Ct.Ar and yield force (*r* = 0.910, FDR-adjusted *p* = 0.005), and pMOI and work to fracture (*r* = 0.780, FDR-adjusted *p* = 0.033) (Figure 5B–E). No significant correlations with post-yield displacement remained after FDR correction. The complete correlation results are provided in Table S1.

## 4. Discussion

In this study, ASC deficiency was associated with remodeling-related changes, deterioration of trabecular microarchitecture and cortical geometry, altered osteocyte LCS organization, and impaired whole-bone mechanical performance in 9-week-old male mice. Correlation analyses further revealed significant associations between cortical geometric parameters and whole-bone mechanical properties. Given the important role of osteocyte LCS in bone mechanosensing and adaptation, the observed LCS alterations may be relevant to the accompanying mechanical phenotype, although osteocyte mechanosensing was not directly assessed in the present study. These findings extend previous work on ASC in osteogenesis and bone repair by defining the broader structural and mechanical phenotype associated with ASC deficiency in the intact skeleton.

Previous studies have mainly focused on the role of ASC in osteoblast differentiation and bone formation. ASC deficiency directly impairs BMP7-induced osteoblast differentiation and attenuates bone formation during defect repair [11]. Subsequent work further demonstrated decreased bone deposition in *Asc*^−/−^ mice following treatment with bone substitute materials [12]. These findings indicate that ASC plays an important role in osteoblast function and bone formation. Our results expand these findings by showing that ASC also affects bone homeostasis under physiological conditions. In the absence of bone injury or bone substitute treatment, *Asc*^−/−^ mice developed significant bone abnormalities. Histological analysis revealed a reduction in osteoblasts and an increase in osteoclasts on the trabecular bone surface. Therefore, in addition to the previously described effects of ASC deficiency on osteogenic activity, increased bone resorption may also contribute to the bone abnormalities observed in *Asc*^−/−^ mice. However, the role of ASC in osteoclast regulation remains unclear. Direct evidence demonstrating that ASC deficiency promotes osteoclastogenesis in vivo is still limited. Nevertheless, inflammasome signaling has been implicated in osteoclast differentiation and pathological bone resorption. Bone matrix-derived signals can activate the NLRP3 inflammasome and promote osteoclast differentiation [23], whereas deficiency of NLRP3 or NLRC4 inflammasomes has been shown to reduce osteoclast differentiation in vitro [24], suggesting that inflammasome signaling participates in the regulation of bone resorption. However, the mechanisms underlying the osteoclast-related changes observed in *Asc^−/−^* mice remain unclear. Further studies are required to determine how ASC influences osteoclast function.

Micro-CT and histological analyses revealed impaired trabecular bone architecture in *Asc*^−/−^ mice. These mice exhibited reduced BV/TV, Tb.N, and Tb.Th, together with increased Tb.Sp. These structural changes were accompanied by altered bone remodeling, characterized by reduced osteoblasts and increased osteoclasts at the trabecular surface, which was further supported by lower serum P1NP and higher TRAcP5b levels. Recent biomimetic bone models have further highlighted the importance of reciprocal interactions between osteoblasts and osteoclasts in shaping the local bone microenvironment [25]. These changes in bone formation- and resorption-related parameters may contribute to the trabecular bone loss and altered trabecular organization observed in *Asc^−/−^* mice. Given that trabecular bone undergoes continuous remodeling and has a high metabolic activity, it is particularly vulnerable to alterations in remodeling balance [26]. Disruption of bone remodeling may impair structural properties of bone by altering trabecular organization and connectivity [27].

ASC deficiency resulted in impaired cortical bone geometry, as indicated by reductions in Tt.Ar, Ct.Ar, Ct.Th, Ps.Pm, pMOI, and *I*_min_. Because *Asc^−/−^* mice also had shorter femora than WT mice, the reductions in size-dependent cortical geometric parameters should be interpreted in the context of reduced overall bone size. Although the micro-CT regions of interest were defined using consistent anatomical locations and fixed analysis lengths, the difference in femoral length remains an important consideration when interpreting these measurements. Altered periosteal geometry may contribute to alterations in cortical size and mechanical properties, as periosteal apposition is important for maintaining bone strength and resistance to fracture [14,28,29]. Compared to trabecular bone, cortical geometry has a more direct impact on the mechanical properties of long bones. In particular, pMOI and *I*_min_ reflect both the amount and distribution of cortical tissue relative to the bending axis, thereby influencing the ability of cortical bone to withstand mechanical loading [14]. Previous biomechanical studies have demonstrated that cortical geometry is closely associated with whole-bone stiffness and load-bearing capacity [30]. Consistent with these structural abnormalities, three-point bending revealed significant reductions in maximum load, stiffness, yield force, and work to fracture in *Asc*^−/−^ mice, whereas post-yield displacement and estimated elastic modulus did not differ significantly between groups. After FDR correction, multiple cortical geometric parameters remained positively correlated with whole-bone mechanical outcomes. Representative strong associations were observed between Ct.Ar and maximum load, Ct.Th and stiffness, Ct.Ar and yield force, and pMOI and work to fracture. These findings support an important contribution of cortical geometry to the reduced whole-bone mechanical performance of Asc^−/−^ femora. Beyond cortical geometry, bone mechanical performance is also influenced by the material properties of the bone matrix [13]. Microindentation analysis revealed reduced hardness, microindentation-derived Young’s modulus, and yield stress in *Asc*^−/−^ mice, providing complementary evidence of altered tissue-level mechanical properties. Because these measurements were obtained from the tibia rather than the femur used for three-point bending, they should not be interpreted as a direct explanation for the femoral mechanical phenotype.

As a critical three-dimensional network embedded within mineralized bone, the LCS not only facilitates nutrient transport and intercellular communication but also serves as an essential structural foundation for mechanical sensing and signal transmission in bone [17,31]. Shear stress generated by interstitial fluid flow within canaliculi activates osteocyte mechanotransduction pathways, thereby regulating adaptive bone remodeling in response to changes in mechanical loading [18,32]. Increasing evidence indicates that the structural integrity of the LCS represents an important determinant of bone quality [33,34,35]. Here, we show that ASC deficiency is associated with enlarged lacunar area, shortened canalicular length, decreased canalicular density, and increased canalicular width, indicating marked alterations in osteocyte LCS architecture. Whether these structural alterations affect osteocyte mechanosensing or adaptive bone remodeling remains to be determined, as functional osteocyte responses were not directly assessed in the present study.

This study has several limitations. First, the sample size was relatively small, with five mice per group used for most skeletal and mechanical analyses. No formal a priori power calculation was performed. The limited sample size should therefore be considered when interpreting these findings, which warrant further evaluation in independent studies. Second, although ASC deficiency was associated with reduced osteoblast-related parameters and increased osteoclast-related parameters, direct assays of osteoblast and osteoclast differentiation or function were not performed. Moreover, using a global ASC-deficient mouse model represents an important limitation of this study. Although this model allowed us to characterize the overall skeletal consequences of ASC loss, it cannot distinguish the specific contributions of ASC in different cell populations or exclude systemic or developmental effects. Functional osteocyte assays were also not performed; therefore, the observed LCS changes should not be interpreted as direct evidence of altered mechanosensing. Future studies using cell type-specific ASC deletion mouse models will be required to define the precise cellular mechanisms underlying the skeletal phenotype associated with ASC deficiency. Third, although microindentation provides information on local tissue-level mechanical properties, bone material quality is also influenced by mineralization, collagen organization, and microdamage. These factors were not directly assessed in the present study and may contribute to the altered material properties observed following ASC deficiency. Intra- and interobserver variability in LCS measurements was not formally assessed. Finally, this study was performed in 9-week-old male mice. Whether the skeletal effects of ASC deficiency vary with age or sex remains to be determined. *Asc*^−/−^ mice also showed higher body weight and shorter femora than WT mice. The basis for the higher body weight in *Asc^−/−^* mice was not investigated in the present study, as body composition and metabolic parameters were not assessed. Because body weight may influence skeletal loading, this difference should be considered when interpreting the skeletal phenotype. In addition, this study cannot establish whether the observed skeletal phenotype develops primarily during bone growth or reflects altered bone remodeling at later stages. Further studies across different developmental stages would help distinguish between these possibilities.

## 5. Conclusions

In this study, we found that ASC deficiency was associated with impaired bone remodeling, deterioration of trabecular and cortical bone architecture, altered osteocyte LCS, and reduced mechanical properties in young adult male mice. Reduced bone formation and increased bone resorption were associated with trabecular bone loss, while cortical geometric parameters were positively correlated with whole-bone mechanical performance. ASC deficiency was also accompanied by reduced tissue material properties. Together, these findings define a multiscale skeletal phenotype associated with ASC deficiency, extending its known role in osteogenesis and bone repair to the structural and mechanical characteristics of the intact skeleton.

## Data Availability

The data supporting the findings of this study are available from the corresponding author upon reasonable request.

## Supplementary Materials

The following supporting information can be downloaded at: https://www.mdpi.com/article/10.3390/bioengineering13101211/s1, Figure S1: Changes in bone formation- and resorption-related parameters in *Asc^−/−^* mice; Table S1: Pearson correlations between cortical geometric parameters and whole-bone mechanical outcomes.
