# Supplementary material for: ASC Deficiency Compromises Skeletal Architecture and Mechanical Competence in Mice
Source: Bioengineering (Basel). 2026 Oct 17;13(10):1211. doi: 10.3390/bioengineering13101211 (PMC13701757; doi:10.3390/bioengineering13101211)
Supplement: Supplementary file 1 [file bioengineering-13-01211-s001.zip › bioengineering-4570675-supplementary.pdf]

# Supplementary materials

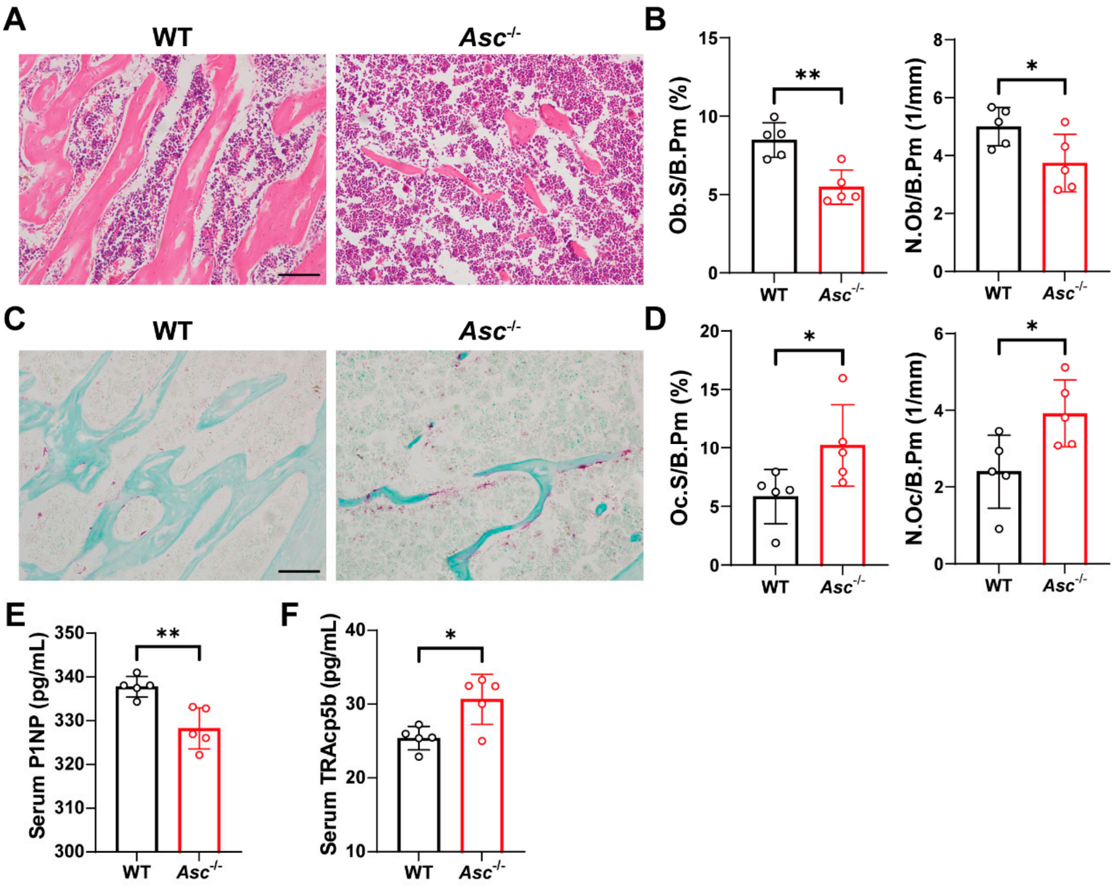


**Figure S1:** Changes in bone formation- and resorption-related parameters in Asc-/- mice. (A) Representative hematoxylin and eosin (H&E)-stained sections of femoral trabecular bone from WT and Asc-/- mice. Scale bar: 100 μm. (B) Quantification of osteoblast surface per bone perimeter (Ob.S/B.Pm) and osteoblast number per bone perimeter (N.Ob/B.Pm). (C) Representative tartrate-resistant acid phosphatase (TRAP)-stained sections of femoral trabecular bone from WT and Asc-/- mice. Scale bar: 100 μm. (D) Quantification of osteoclast surface per bone perimeter (Oc.S/B.Pm) and osteoclast number per bone perimeter (N.Oc/B.Pm). (E) Serum procollagen type I N-terminal propeptide (P1NP) levels. (F) Serum tartrate-resistant acid phosphatase 5b (TRAcP5b) levels. n = 5 mice per group. Individual points represent biological replicates, and data are presented as mean ± SD. Differences between groups were analyzed using a two-tailed unpaired t-test with Welch's correction. $^{*}p < 0.05$, $^{**}p < 0.01$.

**Table S1. Pearson correlations between cortical geometric parameters and whole-bone mechanical outcomes.**

| Cortical geometric parameter | Mechanical outcome | Pearson r | Raw p | BH-FDR-adjusted p |
|---|---|---|---|---|
| Ct.Ar | Maximum load | 0.956 | 0.00002 | 0.00063 |
| Ct.Ar | Stiffness | 0.858 | 0.00150 | 0.00937 |
| Ct.Ar | Yield force | 0.910 | 0.00025 | 0.00506 |
| Ct.Ar | Work to fracture | 0.777 | 0.00825 | 0.03300 |
| Ct.Ar | Post-yield displacement | 0.321 | 0.36652 | 0.41888 |
| Tt.Ar | Maximum load | 0.720 | 0.01879 | 0.04610 |
| Tt.Ar | Stiffness | 0.581 | 0.07825 | 0.12521 |
| Tt.Ar | Yield force | 0.734 | 0.01571 | 0.04581 |
| Tt.Ar | Work to fracture | 0.695 | 0.02554 | 0.04643 |
| Tt.Ar | Post-yield displacement | 0.357 | 0.31125 | 0.37728 |
| Ct.Ar/Tt.Ar | Maximum load | 0.706 | 0.02246 | 0.04610 |
| Ct.Ar/Tt.Ar | Stiffness | 0.700 | 0.02420 | 0.04610 |
| Ct.Ar/Tt.Ar | Yield force | 0.633 | 0.04947 | 0.08603 |
| Ct.Ar/Tt.Ar | Work to fracture | 0.439 | 0.20424 | 0.29178 |
| Ct.Ar/Tt.Ar | Post-yield displacement | 0.084 | 0.81730 | 0.83825 |
| Ct.Th | Maximum load | 0.897 | 0.00043 | 0.00579 |
| Ct.Th | Stiffness | 0.862 | 0.00135 | 0.00937 |
| Ct.Th | Yield force | 0.822 | 0.00352 | 0.01762 |
| Ct.Th | Work to fracture | 0.624 | 0.05369 | 0.08948 |
| Ct.Th | Post-yield displacement | 0.188 | 0.60218 | 0.65100 |
| Ps.Pm | Maximum load | 0.706 | 0.02240 | 0.04610 |
| Ps.Pm | Stiffness | 0.559 | 0.09265 | 0.14255 |
| Ps.Pm | Yield force | 0.730 | 0.01652 | 0.04581 |
| Ps.Pm | Work to fracture | 0.705 | 0.02284 | 0.04610 |
| Ps.Pm | Post-yield displacement | 0.374 | 0.28762 | 0.36848 |
| Ec.Pm | Maximum load | 0.227 | 0.52784 | 0.58649 |
| Ec.Pm | Stiffness | 0.045 | 0.90254 | 0.90254 |
| Ec.Pm | Yield force | 0.324 | 0.36169 | 0.41888 |
| Ec.Pm | Work to fracture | 0.482 | 0.15869 | 0.23510 |
| Ec.Pm | Post-yield displacement | 0.405 | 0.24560 | 0.33876 |
| pMOI | Maximum load | 0.861 | 0.00139 | 0.00937 |
| pMOI | Stiffness | 0.727 | 0.01718 | 0.04581 |
| pMOI | Yield force | 0.854 | 0.00164 | 0.00937 |
| pMOI | Work to fracture | 0.780 | 0.00774 | 0.03300 |
| pMOI | Post-yield displacement | 0.382 | 0.27593 | 0.36791 |
| $I_{min}$ | Maximum load | 0.734 | 0.01562 | 0.04581 |
| $I_{min}$ | Stiffness | 0.368 | 0.29478 | 0.36848 |
| $I_{min}$ | Yield force | 0.701 | 0.02402 | 0.04610 |
| $I_{min}$ | Work to fracture | 0.756 | 0.01149 | 0.04178 |
| $I_{min}$ | Post-yield displacement | 0.089 | 0.80611 | 0.83825 |

**Note:** Pearson correlation analyses were performed using pooled data from WT and $Asc^{-/-}$ mice (n = 10). Raw p-values were adjusted for multiple testing across all 40 correlations using the Benjamini–Hochberg false discovery rate procedure. Elastic modulus was not included in the correlation analysis because it was derived from stiffness and Imin.
